# Native T1 values in discrimination between in acute and chronic myocarditis

**DOI:** 10.1186/1532-429X-16-S1-O62

**Published:** 2014-01-16

**Authors:** Rocio Hinojar Baydes, Eduardo Arroyo Ucar, Lucy Foote, Darius Dabir, Islam Mahmoud, Thomas Jackson, David M Higgins, Tobias Schaeffter, Eike Nagel, Valentina Puntmann

**Affiliations:** 1 King's College London, London, UK; 2 Philips Healthcare, Guilford, UK

## Background

T1 mapping by cardiovascular magnetic resonance (CMR) provides tissue-dependent relaxation times in line with the underlying myocardial composition. In this study, we examined the value of native T1 in differentiation between acute viral myocarditis and chronic stages of healing.

## Methods

Fifty-three patients with a clinical diagnosis of acute viral myocarditis were compared to fifty-six subjects in clinical convalescence. All subjects underwent assessment of myocardial oedema, function and scar by CMR at 1.5 and 3-Tesla. T1 values were measured in midventricular short-axis slice prior to and after administration of 0.2 mmol/kg of gadobutrol.

## Results

**Figure 1:**
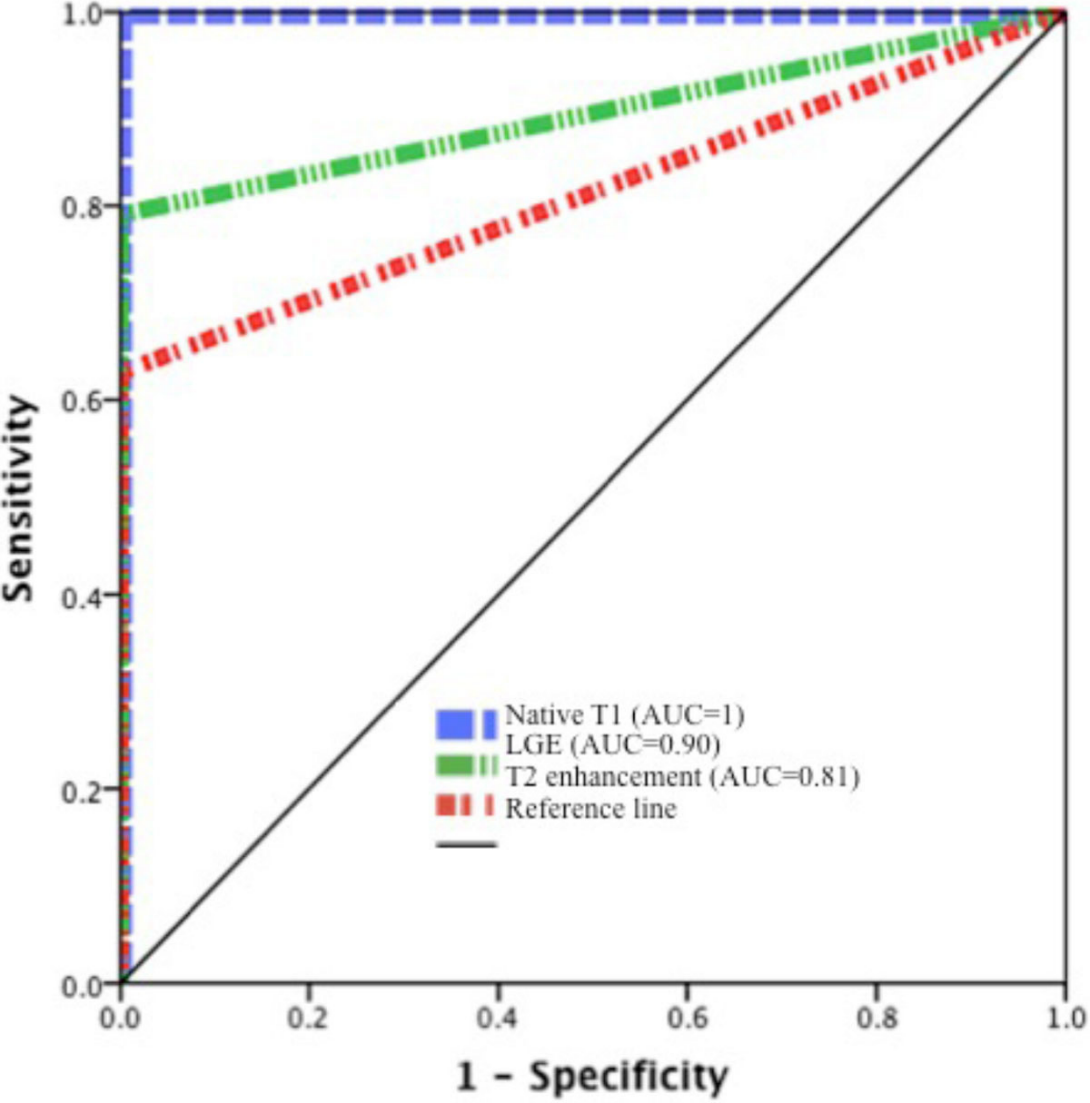
**Native T1 is superior to LGE and T2 enhancement in discrimination between acute and chronic myocarditis**.

Compared to controls (n = 40), native T1 values were increased in both acute and chronic myocarditis at both field strengths (controls vs. acute vs. chronic myocarditis; 1.5T: native T1 (msec): 940 ± 17 vs. 1070 ± 34 vs. 998 ± 28, p < 0.001; 3T: 1045 ± 18 vs. 1182 ± 66 vs. 1094 ± 15, p < 0.001). Native T1 values were significantly higher in acute vs. chronic myocardium (p < 0.001). Native T1 was identified as the independent discriminator between health and disease, as well as acute and chronic myocarditis (Figure 1). Native T1 showed gradual reduction of values between acute and chronic stage of myocardial inflammation.

## Conclusions

We demonstrate that native T1 values can reliably discriminate between patients with acute myocarditis and those subjects in stages of chronic healing at both field strengths. Native T1 values provide a dynamic index of disease activity and progression from acute disease towards clinical resolution.

## Funding

We would like to acknowledge Department of Health via the National Institute for Health Research (NIHR) comprehensive Biomedical Research Centre award to Guy's & St Thomas' NHS Foundation Trust in partnership with King's College London and King's College Hospital National Health Service Foundation Trust. Dr. Rocio Hinojar was supported by the Spanish Society of Cardiology.

